# Neuropeptides Exert Neuroprotective Effects in Alzheimer's Disease

**DOI:** 10.3389/fnmol.2018.00493

**Published:** 2019-01-11

**Authors:** Xin-Yi Chen, Yi-Feng Du, Lei Chen

**Affiliations:** ^1^Department of Physiology and Pathophysiology, Qingdao University, Qingdao, China; ^2^Department of Neurology, Provincial Hospital Affiliated to Shandong University, Jinan, China

**Keywords:** neuropeptide, Alzheimer's disease, ghrelin, neurotensin, pituitary adenylate cyclase-activating polypeptide, neuropeptide Y, substance P, orexin

## Abstract

Alzheimer's disease (AD) is an age-related neurodegenerative disorder characterized by cognitive deficits and neuronal loss. Deposition of beta-amyloid peptide (Aβ) causes neurotoxicity through the formation of plaques in brains of Alzheimer's disease. Numerous studies have indicated that the neuropeptides including ghrelin, neurotensin, pituitary adenylate cyclase-activating polypeptide (PACAP), neuropeptide Y, substance P and orexin are closely related to the pathophysiology of Alzheimer's disease. The levels of neuropeptides and their receptors change in Alzheimer's disease. These neuropeptides exert neuroprotective roles mainly through preventing Aβ accumulation, increasing neuronal glucose transport, increasing the production of neurotrophins, inhibiting endoplasmic reticulum stress and autophagy, modulating potassium channel activity and hippocampal long-term potentiation. Therefore, the neuropeptides may function as potential drug targets in the prevention and cure of Alzheimer's disease.

## Introduction

Alzheimer's disease (AD) is an age-related neurodegenerative disorder which is clinically characterized by cognitive deficits, memory impairment, disorientation, and behavioral issues (Burns and Iliffe, 2009). Commonly the Alzheimer's disease begins in people over the age of 65 years. The neuropathological features in Alzheimer's disease are amyloid plaques, neurofibrillary tangles, and neuronal loss (Tiraboschi et al., 2004). Beta-amyloid peptide (Aβ) oligomers have been reported to be the primary pathogenic forms of Aβ, which change the structure of synapses and eventually disrupt neuronal communication (Lacor et al., 2007). However, the pathogenesis remains unknown.

Neuropeptides are molecules which function as endogenous active substances within central nervous system and peripheral nervous system. Neuropeptides play important roles in a wide range of brain functions, including food intake, metabolism, reproduction, social behaviors, reward, learning and memory, sleep and wakefulness. Recent studies revealed that many neuropeptides including ghrelin, neurotensin, pituitary adenylate cyclase-activating polypeptide (PACAP), neuropeptide Y, substance P, and orexin may be associated with the pathophysiology and potential therapy of Alzheimer's disease. In this article, we review the recent advances about the involvement of neuropeptides in Alzheimer's disease.

## Ghrelin and Alzheimer's Disease

Ghrelin, a 28-amino acid brain-gut peptide, activates the growth hormone secretagogue receptors which are expressed widely in the brain (Guan et al., 1997). Recent studies revealed that the receptor expression is developmental, with a stronger staining in the early stages and a weaker expression in the later stages of development (Lattuada et al., 2013). Ghrelin exerts critical roles in the regulation of energy homeostasis, neuroendocrine and neurodegenerative processes, especially in higher brain functions, such as learning and memory consolidation (Spitznagel et al., 2010; Rak-Mardyla, 2013; Murray et al., 2014; Panagopoulos and Ralevski, 2014; Jiao et al., 2017). Ghrelin is also involved in mitochondrial respiration and neuroprotection, which can be developed as biomarkers or drug targets for prevention and treatment of neurological disorders, including Parkinson's disease, stroke, epilepsy and Alzheimer's disease (dos Santos et al., 2013a; Shi et al., 2014, 2017; Stoyanova, 2014).

Ghrelin has been demonstrated to have a close relationship with Alzheimer's disease. One of the single nucleotide polymorphisms of the ghrelin gene, rs4684677 (Leu90Gln), has been proved to be associated with the onset age of Alzheimer's disease (Shibata et al., 2011). Early study revealed that the level of serum ghrelin is negatively related to several cognitive domains in older adults (Spitznagel et al., 2010). The level of the functional form of ghrelin, acylated ghrelin, is associated with Alzheimer's disease risk factors and mild cognitive impairment (Gahete et al., 2010; Cao et al., 2018). However, a significant reduction of ghrelin mRNA was observed in the temporal gyrus of Alzheimer's disease patients, suggesting the possible involvement of ghrelin in the cognitive deficit of Alzheimer's disease (Gahete et al., 2010).

Administration of ghrelin or ghrelin agonist decreases the level of Aβ and attenuates Alzheimer's disease-related cognitive impairment (Gahete et al., 2010, 2011; Dhurandhar et al., 2013; Kunath et al., 2015). Recent *in vitro* study demonstrated that pre-administration of ghrelin or ghrelin analog protects SH-SY5Y cellular models of Alzheimer's disease against methylglyoxal-induced toxicity and apoptosis, suggesting the potential treatment for neurodegenerative disorders (Cecarini et al., 2016; Popelová et al., 2018). Ghrelin exerts neuroprotective effects through different mechanisms. Central application of acylated ghrelin prevents Aβ-induced impairments of memory and energy and glucose metabolisms, probably through increase of AMPK and GSK phosphorylation and decrease of tau phosphorylation (Kang et al., 2015). Further study revealed that acylated ghrelin also blunts Aβ-induced depression of long-term potentiation (LTP) in hippocampus and therefore prevents impairments of recognition and spatial orientation (Santos et al., 2017). Ghrelin ameliorates neurogenesis impairment in hippocampus and improves memory deficits by prevention of synaptic degeneration including cholinergic fiber loss (Moon et al., 2011, 2014). Recent studies revealed that ghrelin exerts protective effects against Aβ-induced toxicity via preventing superoxide production, calcium elevation and mitochondrial membrane depolarization (Martins et al., 2013; Gomes et al., 2014). In addition, ghrelin restores the proteasome functionality in Alzheimer's disease and thus contributes to the elimination of toxic aggregates (Cecarini et al., 2016). It is known that brain insulin resistance is closely related to the cognitive impairment and neurodegeneration, particularly within Alzheimer's disease. Recently, it is demonstrated that ghrelin increases neuronal glucose uptake and improves tau hyperphosphrylation by activating Akt and GSK-3 beta phosphorylation in cultured hippocampal neurons (Chen et al., 2010).

**Figure 1:**
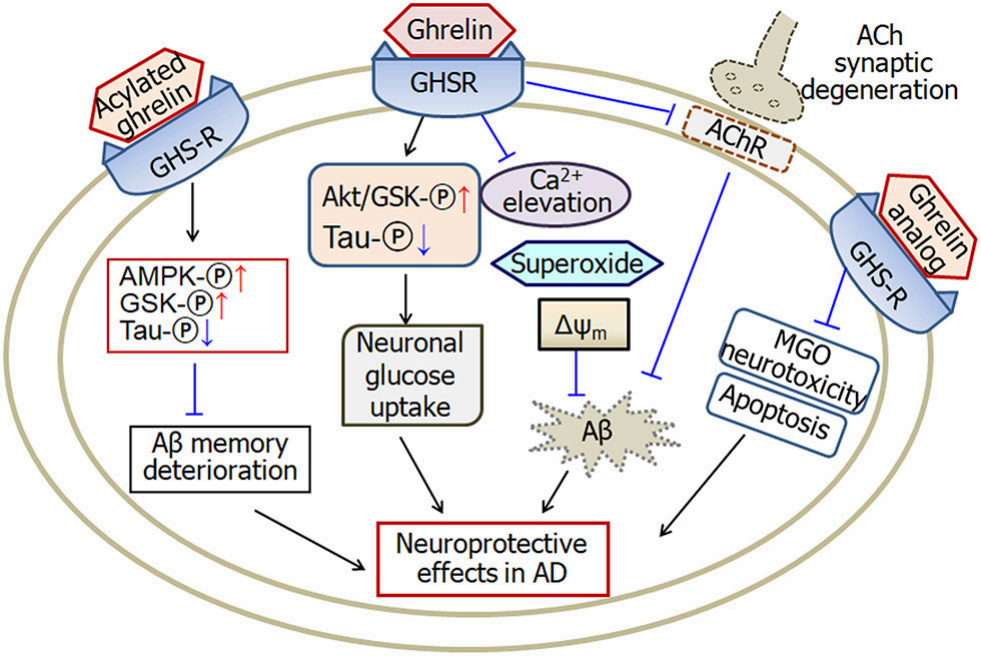
A schematic diagram describing the possible pathways of ghrelin-induced neuroprotective effects in Alzheimer's disease. Ghrelin protects against Aβ-induced neurotoxicity through prevention of calcium elevation, superoxide production and mitochondrial membrane depolarization. Ghrelin also increases neuronal glucose uptake by activating Akt/GSK phosphorylation and improving tau hyperphosphrylation. Moreover, ghrelin prevents cholinergic synaptic degeneration and therefore protects against Aβ-induced memory deficits. Acylated ghrelin improves Aβ-induced deterioration of memory through increase of AMPK and GSK phosphorylation and decrease of tau phosphorylation. Analog of ghrelin protects against MGO-induced neurotoxicity and apoptosis in cellular models of Alzheimer's disease. GHSR, growth hormone secretagogue receptors, also known as ghrelin receptors; Δψm, mitochondrial membrane potential; MGO, methylglyoxal; ACh, acetylcholine; AMPK, adenosine 5′-monophosphate (AMP)-activated protein kinase; GSK, glycogen synthase kinase; AChR, cholinergic receptors; -℗, phosphrylation. The internal and external circles represent the inner and outer leaflets of the cellular membrane. The dotted line in ACh synapse represents the degenerated synapse.

In summary, the levels of ghrelin change in Alzheimer's disease. The functional form of ghrelin, acylated ghrelin, increases in mild cognitive impairment. A single nucleotide polymorphism of ghrelin gene is associated with the onset age of Alzheimer's disease. Ghrelin protects against Aβ-induced neurotoxicity through multiple pathways. Ghrelin prevents calcium elevation, superoxide production and mitochondrial membrane depolarization. Moreover, ghrelin increases neuronal glucose uptake and improves Aβ-induced deterioration of memory through activating AMPK and GSK phosphorylation and decreasing tau phosphorylation. Furthermore, ghrelin prevents cholinergic synaptic degeneration. Therefore, ghrelin is considered as a potential drug in the treatment of Alzheimer's disease (Figure 1).

## Neurotensin and Alzheimer's Disease

**Figure 2:**
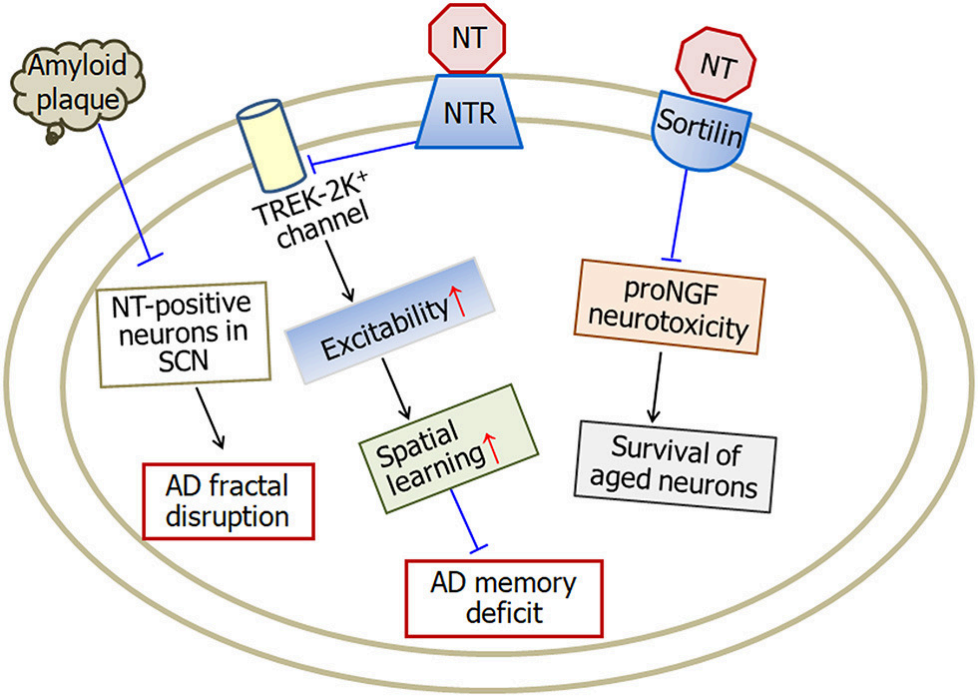
A model illustrating the neuroprotective effects of neurotensin in Alzheimer's disease. Neurotensin increases the excitability of neurons by inhibiting TREK-2K^+^ channel, which therefore improves memory status in Alzheimer's disease mice. Being a sortilin ligand, neurotensin rescues the survival of aged neurons through blocking sortilin-induced proNGF neurotoxicity. In addition, amyloid plaque density in the occipital cortex is negatively associated with neurotensin-positive neurons in the suprachiasmatic nucleus suggesting the involvement of neurotensin in fractal activity disruption in Alzheimer's disease. NT, neurotensin; NTR, neurotensin receptors; SCN, suprachiasmatic nucleus. The internal and external circles represent the inner and outer leaflets of the cellular membrane.

Neurotensin is a tridecapeptide which binds with two neurotensin receptors, type-1 and type-2, in the brain. Neurotensin plays multiple effects in central nervous system and is involved in the pathophysiology of several central nervous system disorders, including schizophrenia (Garver et al., 1991; Kinkead and Nemeroff, 2004), Parkinson's disease (Bissette et al., 1985; Fernandez et al., 1994), as well as Alzheimer's disease (Constantinidis et al., 1983; Struble et al., 1987; Gahete et al., 2010; Xiao et al., 2014). The levels of neurotensin and neurotensin receptors change in several brain regions of Alzheimer's disease patients. Neurotensin influences the formation of senile plaques and therefore is associated with the pathogenesis of Alzheimer's disease through different pathways (Figure 2).

Previous studies revealed a decreased level of neurotensin in the septum (Ferrier et al., 1983), suprachiasmatic nucleus (Hu et al., 2013) and amygdala (Benzing et al., 1990), and a low level of neurotensin receptors in the entorhinal area (Jansen et al., 1990), dentate gyrus (Rowe et al., 2006) and temporal gyrus (Gahete et al., 2010) of Alzheimer's disease. In Alzheimer's disease patients, the amyloid plaque density in the occipital cortex is negatively associated with the neurotensin-positive neurons in the suprachiasmatic nucleus, i.e., the more plaques, the fewer neurotensin-positive neurons, which suggests the involvement of neurotensin in fractal activity disruption in Alzheimer's disease (Hu et al., 2013). The levels of amygdala neurotensin in males are significantly higher than that in females (Biggins et al., 1983), which suggests that the intrinsic function of the amygdala may be associated with hormonal regulation of other sex-dependent mechanism. For example, Skup et al. (2011) reported that the males and the females show different patterns in amygdala volume decline over time. Previous studies revealed that men exhibit greater volumes and neuronal densities in amygdala (Giedd et al., 1996; Goldstein et al., 2001; Witte et al., 2010). Furthermore, the density of neurotensin and acetylcholine containing fibers is reduced dramatically in the regions of amygdala with greatest senile plaques (Benzing et al., 1992, 1993). However, in the case of high plaque non-demented, no significant reduction of neurotensinergic fibers and other neurotransmitter fibers was observed in the amygdala (Benzing et al., 1993).

It is well-known that the entorhinal cortex is a crucial brain region involved in the earliest pathological change of Alzheimer's disease (Mann, 1989; Beach et al., 1997). Recently, Xiao et al. (2014) found that neurotensin persistently increases the spontaneous firing rate of neurons in the entorhinal cortex. This facilitation is mediated by neurotensin type 1 receptor and TREK-2K^+^ channels. Further behavioral studies revealed that activation of neurotensin type 1 receptors enhances spatial learning and therefore improves memory status in APP/PS1 Alzheimer's disease mice model. Recent electrophysiological studies further demonstrated that neurotensin increases glutamate release and spontaneous firing rate of dentate gyrus through both presynaptic and post-synaptic neurotensin receptors, respectively (Zhang et al., 2015, 2016).

It has recently been shown that the precursor form of nerve growth factor (proNGF) forming complex with p75 and sortilin contributes to neuronal death in basal forebrain neurons (Al-Shawi et al., 2007, 2008). Sortilin plays an important role in proNGF-mediated neurotoxicity. As a sortilin ligand, neurotensin blocks the sortilin-induced effects of proNGF and further rescues the survival of old neurons (Al-Shawi et al., 2008).

In summary, the levels of neurotensin and neurotensin receptors decrease in many brain areas of Alzheimer's disease. The amyloid plaque density in the occipital cortex is negatively associated with the neurotensin-positive neurons in the suprachiasmatic nucleus. Neurotensin increases the excitability of entorhinal cortex neurons and therefore improves spatial learning and memory. Furthermore, neurotensin rescues the survival of aged neurons through blocking sortilin-induced proNGF neurotoxicity.

## PACAP and Alzheimer's Disease

**Figure 3:**
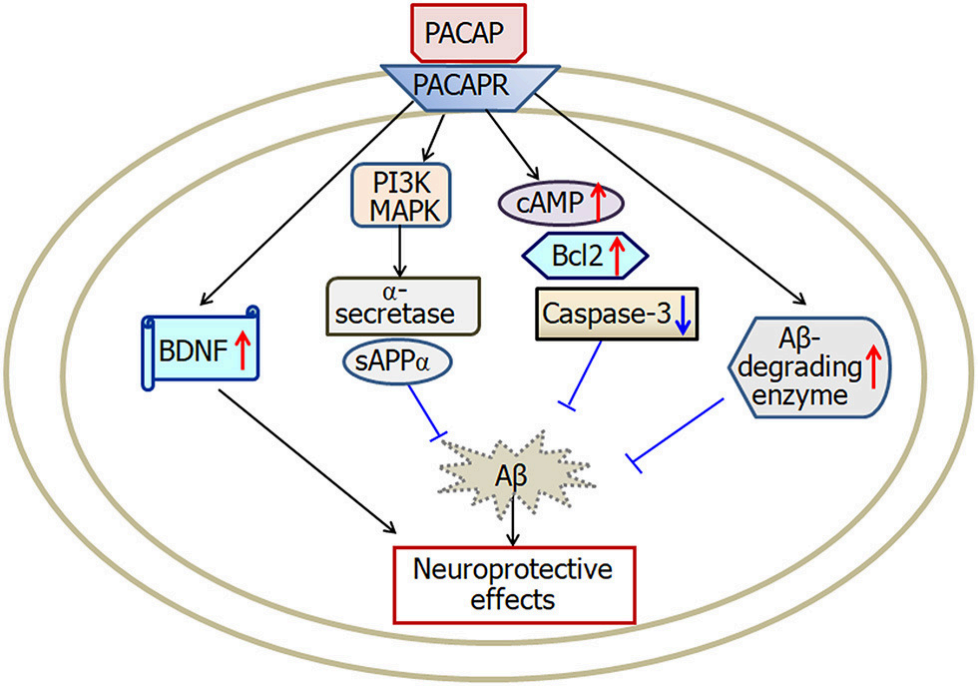
A scheme describing the possible mechanisms of PACAP-induced neuroprotective effects in Alzheimer's disease. PACAP protects against Aβ-induced neurotoxicity by activation of cAMP, BDNF, Bcl-2, Aβ-degrading enzyme and deactivation of caspase-3. Furthermore, PACAP increases α-secretase activation and then enhances secretion of sAPPα through both the MAPK and PI3K pathways. PACAP, pituitary adenylate cyclase-activating polypeptide; PACAPR, pituitary adenylate cyclase-activating polypeptide receptors; BDNF, brain-derived neurotrophic factor. The internal and external circles represent the inner and outer leaflets of the cellular membrane.

PACAP belongs to the superfamily of secretin/glucagon/vasoactive intestinal polypeptide (Miyata et al., 1989). High level of PACAP is expressed in the hypothalamus, hippocampus, cerebellum and several brainstem nuclei (Hannibal, 2002). By activating three types of receptors (PAC1, VPAC1, and VPAC2), PACAP functions as a neurohormone, neurotransmitter, or neurotrophic factor in central nervous system (Lee and Seo, 2014). There is growing evidence (Reglodi et al., 2011) suggests that PACAP is closely associated with the pathology of Alzheimer's disease (Figure 3).

By using three different mouse models of Alzheimer's disease, early study showed downregulation of PACAP genes (Wu et al., 2006). Moreover, the PACAP levels are reduced in brain areas including entorhinal cortex, middle temporal gyrus, superior frontal gyrus, and primary visual cortex in Alzheimer's disease patients (Han et al., 2014a). Further study revealed that the lower PACAP levels are correlated with higher amyloid burden, tau protein, and the declined recognition memory with aging (Han et al., 2014b; An et al., 2017).

It is demonstrated that PACAP exerts neuroprotective effects through multiple mechanisms in Alzheimer's disease patients and mouse models (Vaudry et al., 2004; Rat et al., 2011). Onoue et al. (2002) first demonstrated that PACAP protects against Aβ-induced neuronal toxicity. Treatment with PACAP rescues 80% of Aβ-induced reduction of cell viability through increase of cAMP and deactivation of caspase-3 in PC12 cells. Intranasal application of PACAP increases the levels of brain-derived neurotrophic factor (BDNF) and antiapoptotic Bcl-2 protein. In addition, PACAP also increases the level of Aβ-degrading enzyme (Rat et al., 2011). Therefore, intranasal application of PACAP could be a useful therapeutic approach in treating Alzheimer's disease. It is known that, in the non-amyloidogenic pathway, the α-secretase cleaves the amyloid precursor protein and prevents amyloid plaque formation (Postina, 2012). Activation of PAC1 receptors increases APPs alpha secretion and enhances α-secretase cleavage of APP. Furthermore, both the MAPK and PI3K pathways are involved in PACAP-mediated α-secretase activation (Kojro et al., 2006).

Ginsenoside is a major component of the traditional herb ginseng which has been proved to exert neurotrophic and neuroprotective effects via preventing neuronal degeneration. Ginsenosides Rh2 increases the gene expression of PACAP in astrocytes of the brain and therefore ameliorates Aβ-induced growth inhibition of astrocytes (Shieh et al., 2008).

In summary, the level of PACAP decreases in brain areas of Alzheimer's disease patients and mouse models, which is correlated with higher amyloid burden, tau protein, and the declined recognition memory. PACAP protects against Aβ-induced neuronal toxicity through activation of cAMP, Bcl2, BDNF, Aβ-degrading enzyme, and deactivation of caspase-3. In addition, PACAP enhances α-secretase cleavage of APP via both MAPK and PI3K pathways.

## Neuropeptide Y and Alzheimer's Disease

**Figure 4:**
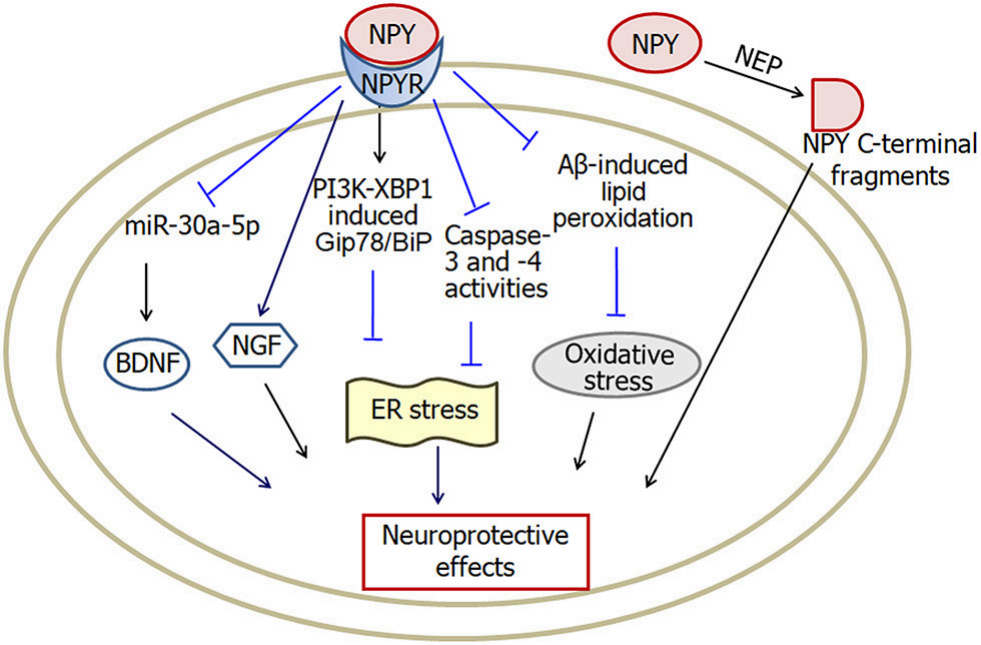
A model showing the possible pathways of neuropeptide Y-induced neuroprotective effects in Alzheimer's disease. Neuropeptide Y inhibits Aβ-induced lipid peroxidation and prevents intracellular oxidative stress. Activation of PI3K-XBP1 pathway may also be involved in neuropeptide Y-induced neuroprotection against endoplasmic reticulum stress. Moreover, both NGF and BDNF are involved in neuropeptide Y-induced neuroprotective effects. In addition, NEP cleaves neuropeptide Y into C-terminal fragments, which protect against the neurodegenerative pathology in Alzheimer's disease. NPY, neuropeptide Y; NPYR, neuropeptide Y receptors; NEP, neutral endopeptidase; ER, endoplasmic reticulum; BDNF, brain-derived neurotrophic factor; NGF, nerve growth factor. The internal and external circles represent the inner and outer leaflets of the cellular membrane.

Neuropeptide Y is a 36-amino acid neuropeptide. Neuropeptide Y receptors are classified into five subtypes: Y1, Y2, Y4, Y5, and Y6 (Hsieh et al., 2013; Mittapalli and Roberts, 2014; Pérez-Fernández et al., 2014). Neuropeptide Y plays functions associated with modulation of food ingestion, mood, learning and memory (dos Santos et al., 2013b) and also plays an important role in neuroprotection against neurodegenerative diseases (Figure 4).

The expression of neuropeptide Y changes under neurodegenerative diseases including Alzheimer's disease (Duarte-Neves et al., 2016). An anomalous high expression level of neuropeptide Y with aging was detected in the hippocampal circuits of mouse model of Alzheimer's disease (Diez et al., 2003; Krezymon et al., 2013). Recently, Mahar et al. (2017) reported that the number of neuropeptide Y immunoreactive hippocampal interneurons reduces in presymptomatic TgCRND8 Alzheimer's disease mouse model.

Neuropeptide Y has neuroprotective effects in Alzheimer's disease (Croce et al., 2011, 2012, 2013; Angelucci et al., 2014; Duarte-Neves et al., 2016; Spencer et al., 2016). Intracerebroventricular application of neuropeptide Y prevents Aβ_1−40_-induced depressive-like symptoms and spatial memory impairments through Y2 receptors. Neuropeptide Y produces the neuroprotection through inhibition of Aβ-induced lipid peroxidation, indicating the involvement of prevention of intracellular oxidative stress (dos Santos et al., 2013b). Moreover, neuropeptide Y induces protective effects against endoplasmic reticulum stress-mediated cell loss through activation of PI3K-XBP1-induced Gip78/BiP pathway as well as inhibition of caspase-3 and caspase-4 activities (Lee et al., 2018). Neuropeptide Y-induced neuroprotective effects are also associated with the production of neurotrophin family (Croce et al., 2011, 2012, 2013; Angelucci et al., 2014). Pretreatment with neuropeptide Y protects neurons against Aβ neurotoxicity which is accompanied by an increased intracellular level of NGF (Croce et al., 2012) and BDNF (Croce et al., 2013). Moreover, decrease of miR-30-5p (a membrane of miR-30a family regulating BDNF tuning expression) levels is involved in neuropeptide Y-induced modulation of BDNF.

The neutral endopeptidase (NEP) neprilysin is an Aβ degrading enzyme which is therefore involved in the pathogenesis of Alzheimer's disease. However, NEP has also been proved to cleave neuropeptide Y into C-terminal fragments. The NEP-produced C-terminal fragments of neuropeptide Y attenuate the neurodegenerative process in both transgenic Alzheimer's disease mice and Aβ treated human neurons (Rose et al., 2009). It is well-known that Aβ accumulation, senile plaque formation and neuronal dysfunction contribute to the cognitive declination in Alzheimer's disease. However, weight loss is an early sign of Alzheimer's disease. It is reported that high level of Aβ potentially devastates hypothalamic arcuate neuropeptide Y neurons and downregulates the leptin state in the early disease process, which may lead to the weight loss (dos Santos et al., 2013b; Ishii et al., 2014).

In summary, the level of neuropeptide Y increases significantly in hippocampus of mouse Alzheimer's disease models. Neuropeptide Y exerts neuroprotective effects through multiple pathways. Neuropeptide Y mitigates endoplasmic reticulum stress-induced neuronal cell death through activation of PI3K-XBP1-induced Gip78/BiP pathway and inhibition of caspase-3 and caspase-4 activities. Furthermore, neuropeptide Y suppresses oxidative stress via inhibition of Aβ-induced lipid peroxidation. In addition, neuropeptide Y plays neuroprotection via increasing the levels of BDNF and NGF.

## Substance P and Alzheimer's Disease

The tachykinin family includes substance P, neurokinin A and neurokinin B. By activating neurokinin receptors, tachykinin plays important roles including pain, depression, nausea and emesis (Mantyh, 2002). In addition, tachykinin protects against the neurotoxic processes of Alzheimer's disease by multiple pathways (Severini et al., 2016).

Early studies revealed that the expression of substance P changes in different brain regions of Alzheimer's disease. The level of substance P decreases in the cortex, hippocampus and striatum in Alzheimer's disease patients and animal models (Bouras et al., 1990; Quigley and Kowall, 1991; Nag et al., 1999; Ahmed et al., 2004), but increases in the pallidum and substantia nigra (Bouras et al., 1990). However, Willis et al. (2007) showed that in old age of Alzheimer's disease transgenic mice, the substance P-immunoreactivity exists in astrocytes of hippocampus and thalamus. In late onset Alzheimer's disease patients, the level of substance P in cerebrospinal fluid increases significantly (Rösler et al., 2001). Furthermore, the increased level of substance P is positively associated with the level of Aβ_1−42_ in Alzheimer's disease patients (Johansson et al., 2015). In addition to the change of substance P, the activity of neuropeptidases to metabolize substance P also alters in Alzheimer's disease. Waters and David (1995) demonstrated that the activity of neuropeptidases is decreased in the temporal cortex of senile dementia of the Alzheimer's disease, which therefore increases the metabolic half-life of substance P.

**Figure 5:**
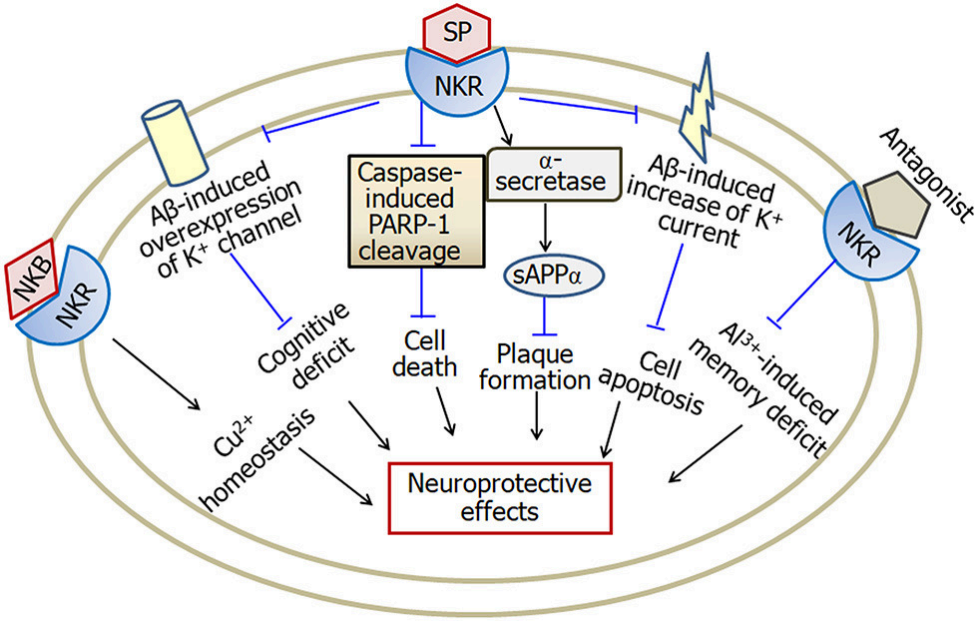
A model describing the multiple effects of substance P in Alzheimer's disease. Substance P inhibits Aβ-induced overexpression of K^+^ channel and Aβ-induced increase of K^+^ current, and therefore attenuates cognitive deficit and apoptosis in Alzheimer's disease. Furthermore, substance P exerts neuroprotective effects through inhibition of caspase-3-induced PARP-1 cleavage and enhancement of α-secretase activity. Neurokinin B plays a role in copper homeostasis. However, substance P receptor antagonist attenuates aluminum-induced spatial memory deficit probably through blockade of substance P-mediated neuroinflammation. SP, substance P; NKR, neurokinin receptors; PARP-1, poly ADP-ribose polymerase-1; sAPPα, soluble amyloid precursor protein α; NKB, neurokinin B; Cu, copper; Al, aluminum. The internal and external circles represent the inner and outer leaflets of the cellular membrane.

Substance P exerts neuroprotective effects in a variety of *in vitro* and *in vivo* studies (Figure 5). Potassium channel dysfunction is a possible mechanism underlying the pathophysiology of Alzheimer's disease. Some kinds of voltage-gated potassium channels are involved in substance P-induced neuroprotective effects. Application of substance P prevents Aβ-induced impairment of cognitive processes through inhibition of Aβ-induced overexpression of potassium channel subunits (Campolongo et al., 2013), as well as Aβ-induced enhancement of A-type K^+^ currents (Pieri et al., 2010). Substance P also protects cerebellar granule cells against Aβ-induced apoptosis through inhibition of caspase-3-induced PARP-1 cleavage (Pieri et al., 2010). In addition, substance P plays non-amyloidogenic effect through decreasing Aβ_1−42_, increasing sAPPα and α-secretase activity (Marolda et al., 2012). Aβ_25−35_ reduces the expression of substance P in hippocampus before the neuronal loss of Alzheimer's disease. Memantine, a non-competitive NMDA receptor antagonist, attenuates Aβ_25−35_-induced decrease of substance P (Arif et al., 2009). Furthermore, in ibotenic acid-treated Alzheimer's disease model, memantine treatment recovers the decreased substance P expression (Ahmed et al., 2004). Recently, Fernandes et al. (2018) reported that a substance P receptor antagonist attenuates aluminum-induced spatial memory deficit probably through blockade of substance P-mediated neuroinflammation. It is known that the dysfunction of metal ions, such as copper is a feature of Alzheimer's disease. Neurokinin B protects against copper-induced calcium channel opening (Russino et al., 2013), as well as the synaptic homeostasis (Grosas et al., 2014).

In summary, the levels of substance P decrease in brain regions including cortex and hippocampus, but increase in cerebrospinal fluid of late onset Alzheimer's disease patients. Substance P exerts neuroprotective effects through inhibition of Aβ-induced over- expression and activity of K^+^ channel. Furthermore, substance P inhibits caspase-3-induced PARP-1 cleavage and increases α-secretase activity, and is therefore involved in neuroprotection in Alzheimer's disease.

## Orexin and Alzheimer's Disease

Orexin is a hypothalamic neuropeptide which plays an important role in maintaining wakefulness. It is well-known that sleep disturbances are common clinical symptom in neurodegenerative disorders. Recently people focus on the involvement of orexinergic system in the pathophysiology, especially sleep disturbances, of Alzheimer's disease (Ferini-Strambi, 2014; Malkki, 2014; Liguori, 2017; Liguori et al., 2017). Early morphological study revealed that the orexinergic neurons are reduced significantly in postmortem hypothalamus of Alzheimer's disease patients (Fronczek et al., 2012). However, it is demonstrated recently that the level of orexin-A in cerebrospinal fluid is higher in Alzheimer's disease patients which is positively associated with Alzheimer's disease biomarkers including Aβ and Tau (Liguori et al., 2016; Osorio et al., 2016; Gabelle et al., 2017). In both Alzheimer's disease patients and Aβ_42_ treated-SH-SY5Y cells, amyloid deposition and tau phosphorylation decrease the expression of orexin-1 and orexin-2 receptors (Davies et al., 2015). *In vivo* microdialysis revealed that infusion of orexin-A increases the amount of Aβ in brain interstitial fluid, while orexin receptor antagonist suppresses Aβ levels and reduces Aβ plaque deposition in the cortex (Kang et al., 2009). In Aβ-treated microglial cells, both orexin-A and orexin-B reduce the uptake of Aβ through downregulation of phagocytosis regulating molecules including PI3K, Akt and p38-MAPK. In addition, orexins suppress autophagosome-lysosome fusion and lead to impaired Aβ degradation (An et al., 2017). Furthermore, the orexin receptors 2 gene, rs2653349 polymorphism, is likely to be a risk factor of Alzheimer's disease (Gallone et al., 2014). In APP/PS1 transgenic Alzheimer's disease mice, orexin gene knock out markedly decreases the amount of Aβ pathology, while rescue of orexinergic neurons increases the amount of Aβ pathology in the brain (Roh et al., 2014). Recent study revealed that orexinergic system modulates both the hippocampal clock and clock-controlled-genes, Bace1 and Bace2. Both the two genes are correlated with the production of Aβ (Ma et al., 2016).

In addition to the inhibition of Aβ uptake and degradation in microglial cells (An et al., 2017), orexin may also exert neuroprotective effects. Orexinergic neuron degeneration impairs long-term social memory in mice (Yang et al., 2013). Intracerebroventricular microinjection of orexin-A improves memory in SAMP8 Alzheimer disease mice (Jaeger et al., 2002). Orexin receptors have been demonstrated to exert neuroprotective effects in Alzheimer's disease via heterodimerization with GPR103. ERK is a key molecule in the prevention of neurodegeneration. Treatment with orexins induces ERK_1/2_ phosphorylation suggesting the neuroprotection of the heterodimerization. Microarray showed that orexin-A augments NF-κB signaling and orexin-B up-regulates PI3K-Akt and Jak-STAT signaling, respectively (Davies et al., 2015).

The hippocampus is a crucial brain region that plays important roles in learning and memory. Intra-hippocampal administration of orexin-A attenuates pain-induced impairment of learning and memory (Raoof et al., 2015), while blocking orexin receptors in hippocampal CA1 regions impairs spatial memory retrieval (Akbari et al., 2006, 2008). It has been demonstrated that activation of cholinergic transmission increases the neuronal excitability of CA1 pyramidal neurons and therefore enhances hippocampus-dependent learning in Alzheimer disease animal model (Disterhoft and Oh, 2006). Moreover, decreasing the firing rate of CA1 pyramidal neurons by potassium channel activator impairs associative learning in rats (McKay et al., 2012). Therefore, our recent study that orexin-A increases the firing rate of hippocampal CA1 neurons may provide direct *in vivo* electrophysiological evidence for the possible involvement of orexin-A in Alzheimer disease (Chen et al., 2017).

**Figure 6:**
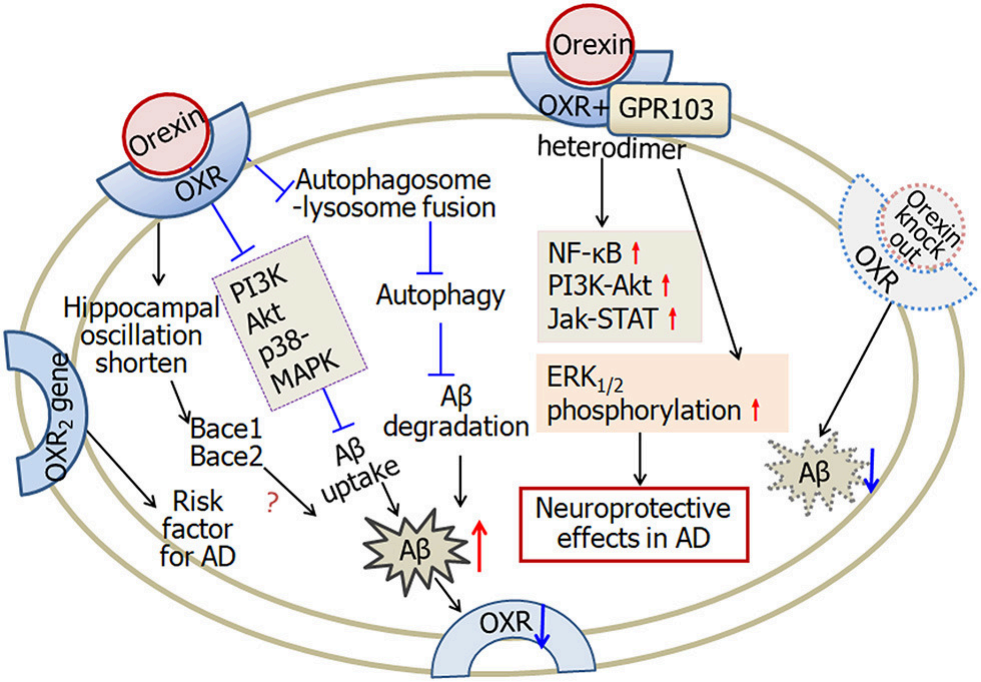
A scheme describing the complicated effects of orexin in Alzheimer's disease. Firstly, in microglial cells, orexin suppresses autophagosome-lysosome fusion process, leading to impaired Aβ degradation. Furthermore, orexin suppresses Aβ uptake through downregulating phagocytosis regulating molecules, such as PI3K, Akt, and p38-MAPK. Aβ-plaque formation and tau hyper-phosphorylation decrease the expression of orexin receptors in Alzheimer's disease. Secondly, orexin receptors and GPR103 form functional heterodimers. Orexin augments NF-κB, PI3K-Akt, Jak-STAT signaling and induces ERK_1/2_ phosphorylation, and therefore is involved in neuroprotective functions. Finally, one of the orexin receptor 2 gene is likely a risk factor for Alzheimer's disease. In APP/PS1 Alzheimer's disease mice, orexin gene knock out decreases the amount of Aβ. Orexin modulates the hippocampal oscillation and the expression of clock-controlled-genes, Bace1 and Bace2, which are associated with the production of Aβ. OXR, orexin receptors; AD, Alzheimer's disease. The internal and external circles represent the inner and outer leaflets of the cellular membrane.

In summary, Aβ deposition and tau phosphorylation decrease the expression of orexin and the receptors in hypothalamus, while the levels of orexins in cerebrospinal fluid increase in Alzheimer's disease patients. In microglial cells, orexins increase Aβ level through suppression of Aβ uptake and degradation. However, in cells forming OXRs and GPR103 heterodimers, orexins exert neuroprotective effects through upregulation of NF-κB, PI3K-Akt, Jak-STAT signaling, as well as ERK_1/2_ phosphorylation. Figure 6 illustrated the complicated effects of orexin in Alzheimer's disease.

## Conclusion

In this review article, we provide a description of recent advances of neuropeptides including ghrelin, neurotensin, PACAP, neuropeptide Y, substance P and orexin in Alzheimer's disease. Based on current studies, the levels of these neuropeptides and their receptors change in Alzheimer's disease (Table 1). Neuropeptides exert significant neuroprotective effects against Aβ-induced neuronal toxicity (Table 2). Multiple intracellular mechanisms, including activation of α-secretases and Aβ-degrading enzyme, production of neurotrophins, increase of neuronal glucose uptake, upregulation of NF-κB, PI3K-Akt, MAPK, Jak-STAT, ERK_1/2_ phosphorylation and downregulation of caspase-3, inhibition of endoplasmic reticulum stress and autophagy, modulation of LTP and potassium channel activity, appear to be involved in neuropeptide-induced neuroprotection in Alzheimer's disease. As the levels of neuropeptides and the receptors may change before Aβ deposition and neuronal loss, as well as positively/negatively correlate with cognitive impairment, future studies would focus on detection of neuropeptides as biomarkers in early diagnosis and treatment evaluation of Alzheimer's disease. Furthermore, for the notable neuroprotective effects, further researches are needed to explore the potential use of neuropeptides, especially via convenient administration method like intranasal application, in the prevention and cure of Alzheimer's disease.

**Table 1:** Changes in the level of neuropeptides and receptors in Alzheimer's disease.

**Neuropeptides**	**Receptors**	**Levels**	**Biological sample**	**State of disease**		**References**
				**Human**	**Animal models**	
Ghrelin mRNA		↓	Temporal gyrus	AD patient		Gahete et al., 2010
Acylated ghrelin		↑	Serum	MCI patient		Cao et al., 2018
	GHS-R1a	↓	Temporal gyrus	AD patient		Gahete et al., 2010
	GHS-R1b	↑	Temporal gyrus	AD patient		Gahete et al., 2010
Neurotensin		↓	Amygdala	AD patient		Benzing et al., 1990, 1992, 1993
Neurotensin		↓	Septum	AD patient		Ferrier et al., 1983
Neurotensin mRNA		↓	Temporal gyrus	AD patient		Gahete et al., 2010
	NTSR1, NTSR2	↓	Temporal gyrus	AD patient		Gahete et al., 2010
	NTSR	↓	Entorhinal area	AD patient		Jansen et al., 1990
	NTSR	↓	Dentate gyrus, SNc, VTA, PVNh		Aged (24–25 months) rats with or without memory impairment	Rowe et al., 2006
PACAP gene		↓	Cortex		APP/PS-1 (18 months) and Tg2576/PS-1 (12 months) mice with Aβ deposition	Wu et al., 2006
			Temporal cortex	AD patients		
PACAP		↓	Entorhinal cortex, middle temporal gyrus, superior frontal gyrus, primary visual cortex	AD patient		Han et al., 2014a
PACAP		↓	Hippocampus, cortex around hippocampus		hAPP mice in each age group	Han et al., 2017
Neuropeptide Y		↑	Dentate gyrus		Tg2576 mice (18 months)	Krezymon et al., 2013
Neuropeptide Y		↑	Hippocampus, cortex		APP23 mice (27 months) with Aβ plaques	Diez et al., 2003
Neuropeptide Y		↓	Hippocampal interneurons		TgCRND8 mice (1 month before amyloid deposition)	Mahar et al., 2017
Substance P		↓	Dentate gyrus	AD patient		Quigley and Kowall, 1991
Substance P		↓	Cortex, hippocampus		Rats with Aβ infusion	Nag et al., 1999
Substance P		↓	Cortex, hippocampus	AD patient		Bouras et al., 1990
Substance P		↑	Pallidum, substantia nigra	AD patient		Bouras et al., 1990
Substance P		↑	Astrocytes in hippocampal formation and thalamus		TgAPP751 mice (12 months with Aβ plaques)	Willis et al., 2007
Substance P		↑	CSF	Late onset AD patient		Rösler et al., 2001; Johansson et al., 2015
Orexin		↓	Hypothalamus	AD patient		Fronczek et al., 2012
Orexin		↑	CSF	AD patient		Liguori et al., 2016; Osorio et al., 2016; Gabelle et al., 2017
Orexin precursor gene		↑	Hypothalamus		APP/PS1dE9 mice (12–15 months)	Ma et al., 2016
	OX1R, OX2R	↓	Hippocampus, Aβ42 treated SH-SY5Y cells	AD patient	Aβ plaques and tau phosphorylation	Davies et al., 2015

*AD, Alzheimer's disease; CSF, cerebrospinal fluid; GHS-R, ghrelin G-protein coupled receptor; MCI, mild cognitive impairment; NTSR, neurotensin receptor; OXR, orexin receptor; PACAP, pituitary adenylate cyclase-activating polypeptide; PVNh, paraventricular nucleus of the hypothalamus; SNc, substantia nigra zona compacta; VTA, ventral tegmental area*.

**Table 2:** Neuroprotective effects and possible mechanisms of neuropeptides in Alzheimer's disease.

**Neuropeptides**	**Reagents or treatments**	**Concentrations and times used**	**Neuroprotective effects**	**Mechanisms**	**Cellular and animal models**	**References**
Ghrelin	Ghrelin agonist: LY444711	30 mg/kg/day (in a chocolate pill) for 4 months	Improves cognition	Reduces Aβ and microglial inflammation in dentate gyrus; Impairs glucose tolerance immediately	Tg APPSwDI mice	Dhurandhar et al., 2013; Kunath et al., 2015
	Ghrelin	0.1 and 1 μM for 24 h	Growth-promoting effect on neuronal cells	Induces GHS-R1 expression; Activate the proteasome; Deregulates autophagy	APP-transfected SH-SY5Y cells	Cecarini et al., 2016
	Acyl-ghrelin or DES-acyl ghrelin	0.2 nmol/h for 3 weeks	Reverses impairments of cognition and energy and glucose metabolism	Suppresses Aβ deposition; Increases the phosphorylation of AMPK and GSK, decreases the phosphorylation of tau	Rats with i.c.v infusion of Aβ	Kang et al., 2015
	Acyl-ghrelin	0.3 mg/kg i.p. daily for 7 days	Prevents impairment of recognition and spatial orientation	Blunts Aβ-induced depression of LTP in hippocampus	Mice with i.c.v infusion of Aβ	Santos et al., 2017
	Ghrelin	80 μg/kg i.p. daily for 7 days	Rescues memory deficits	Decreases microgliosis in hippocampus; Attenuates hippocampal neuronal loss; Prevents synaptic degeneration including cholinergic fiber loss	Intrahippocampal injection of AβO	Moon et al., 2011
	Ghrelin	80 μg/kg i.p. every 2 days for 30 days	Ameliorates neurogenesis impairment in hippocampus	N/A	5XFAD mice	Moon et al., 2014
	Ghrelin	0.1–0.5 μM for 24 h	Improves cell survival	Reduces superoxide production and mitochondrial membrane depolarization; Prevents GSK 3β	AβO treated primary hippocampal neurons and hypothalamic N42 cell line	Martins et al., 2013; Gomes et al., 2014
	Ghrelin	10 nM for 1 h	Augments neuronal glucose uptake	Decreases tau phosphoralation; Increases Akt/GSK phosphorylation	Primary hippocampal neurons treated with glucose	Chen et al., 2010
Neurotensin	Neurotensin or NTS1 agonist: PD149163	0.25 μM microinjection or bath application	Improves spatial learning and memory; Increases firing rate of AP in stellate neurons of EC	Inhibits TREK-2K channels via PLC/PKC pathway	APP/PS1 mice	Xiao et al., 2014
	Neurotensin	40 μM for 2 h	Rescues the survival of aged neurons	Blocks sortilin-mediated neurotoxic role of proNGF in old age; Increases proNGF expression in frontal cortex and hippocampus	Aged mice BFN neurons; AD patients	Al-Shawi et al., 2007, 2008
PACAP	PACAP38	10 μg daily for 3 months intranasal	Rescues impaired recognition; Stimulates the non-amyloidogenic processing of APP; Reduces Aβ_40_ and Aβ_42_	Enhances gene expression of α-secretases; Enhances Aβ-degrading enzyme neprilysin; Increases PACAP and PAC1 expression; Increases expression of BDNF; Increases expression of antiapoptotic Bcl-2	APP[V717I]- transgenic mice; SK-N-MC PAC1 cells (BDNF study)	Rat et al., 2011
	PACAP27	1 nM for 72 h	Rescues Aβ-induced cell death	Increases cAMP formation; Decreases caspase-3 activity	Aβ-treated PC12 cells	Onoue et al., 2002
	PACAP27	1 μM for 4 h 300 nM for 4 h	Enhances secretion of sAPPα	Stimulates MAPK pathway and PI3K	SK-N-MC cells; HEK 293 cells	Kojro et al., 2006
Neuropeptide Y	Neuropeptide Y	0.0234 μM/μL, i.c.v.	Prevents depressive-like behavior and spatial memory deficits	Blunts Aβ-induced increase in lipid peroxidation in hippocampus and prefrontal cortex	Mice treated with i.c.v. Aβ_1−40_	dos Santos et al., 2013b
	Neuropeptide Y	100 nM for 12h	Attenuates ER stress-induced cell death	Decreases caspase-3 and−4 activities; Suppresses the activation of three major ER stress sensors; Activates PI3K-XBP1 pathway	SK-N-SH cells; Mouse cortical neurons	Lee et al., 2018
	Neuropeptide Y	0.5, 1, and 2 μM for 24 h	Rescues Aβ-induced cell death	Increases NGF synthesis and restores NGF release; Increases the levels of BDNF via inhibiting miR-30-5p	Aβ_25−35_-treated SH-SY5Y cells; Aβ_25−35_-treated primary cortical neurons	Croce et al., 2012, 2013
	Amidated NPY CTFs (NPY21–36 and 31–36)	120 μM i.c.v. for 28 days *in vivo*; 10 nM for 24 h *in vitro*	Ameliorates neurodegenerative pathology; Protects human neuronal cultures	NPY CTFs generated during NEP-mediated proteolysis exerts neuroprotective effects *in vivo*	APP tg mice; Aβ_1−42_-treated primary human cortical neurons	Rose et al., 2009
Substance P	Substance P	50 mg/kg, i.p. daily for 7 days	Prevents cognitive impairments	Reduces Aβ-induced overexpression of Kv1.4 in hippocampus and cerebral cortex	Rats with i.c.v infusion of Aβ_25−35_	Campolongo et al., 2013
	Substance P	200 nM	Reverses cell death; Reverses Aβ-induced increase of *I*_KA_ current	Inhibits caspase-3-induced PARP-1 cleavage through Akt-dependent mechanism; Prevents Aβ-induced upregulation of Kv4.2 and Kv4.3	Aβ-treated rat cerebellar granule cells	Pieri et al., 2010
	Substance P	200 nM	Reverses K^+^-induced apoptotic cell death and amyloidogenic processing of APP	Enhances α-secretase activity; Increases sAPPα; Reduces Aβ_1−42_	Rat cerebellar granule cells	Marolda et al., 2012
Orexin	Orexin-A Dual orexin receptor antagonist: almorexant	1.5 pM i.c.v for 6 h 13.9 nM i.c.v for 24 h i.p. for 8 weeks	Increases ISF Aβ levels; Suppresses ISF Aβ levels; Reduces Aβ plaque deposition in the cortex	N/A	Human APP transgenic (Tg2576) mice; APPswe/PS1dE9 mice	Kang et al., 2009
	Orexin-A; Orexin-B	1 μM for 24 h	Suppresses the uptake of Aβ; Reduces the degradation of Aβ	Downregulates phagocytosis regulating molecules: PI3K, Akt and p38-MAPK; Interferes autophagosome- lysosome fusion, and then suppresses autophogic flux	Aβ-treated BV2 microglial cells	An et al., 2017
	Orexin-A; Orexin-B	100 nM for 24 h 100 nM for 1 h	Augments NF-κB signaling (OXA); Up-regulates PI3K-Akt and Jak-STAT signaling (OXB); Induces ERK_1/2_ phosphorylation	NF-κB regulates cell survival, neurogenesis, learning and memory; PI3K-Akt is involved in neuroprotective functions; ERK is a key molecule in prevention of neurodegeneration	SH-SY5Y cells treated with retinoic acid (microarray); HEK293 cells with OXRs and GPR103 form functional heterodimers	Davies et al., 2015

*AβO, amyloid-β oligomers; AD, Alzheimer's disease; AMPK, adenosine 5′-monophosphate (AMP)-activated protein kinase; AP, action potential; APP, amyloid precursor protein; BDNF, brain-derived neurotrophic factor; BFN, basal forebrain nuclei; CTFs, C-terminal fragments; EC, entorhinal cortex; ER, endoplasmic reticulum; GHS-R1, growth hormone secretagogue receptor type 1; GSK, glycogen synthase kinase; i.c.v., intracerebroventricular; I_KA_ current, A-type K^+^ current; ISF, interstitial fluid; LTP, long-term potentiation; MAPK, mitogen-activated protein kinase; NEP, neutral endopeptidase; NGF, nerve growth factor; NPY, neuropeptide Y; NTS1, neurotensin receptor 1; OXA, orexin-A; OXB, orexin-B; PACAP, pituitary adenylate cyclase-activating polypeptide; PARP-1, poly ADP-ribose polymerase-1; PI3K, phosphatidylinositol 3-kinase; sAPPα, soluble amyloid precursor protein α; SK-N-MC PAC1 cells, human neuroblastoma SK-N-MC cells overexpressing PAC1 receptor*.

## Author Contributions

X-YC wrote the manuscript. Y-FD and LC revised the manuscript.

### Conflict of Interest Statement

The authors declare that the research was conducted in the absence of any commercial or financial relationships that could be construed as a potential conflict of interest.
